# Altered Expression of CD300a Inhibitory Receptor on CD4+ T Cells From Human Immunodeficiency Virus-1-Infected Patients: Association With Disease Progression Markers

**DOI:** 10.3389/fimmu.2018.01709

**Published:** 2018-07-23

**Authors:** Joana Vitallé, Iñigo Terrén, Leire Gamboa-Urquijo, Ane Orrantia, Laura Tarancón-Díez, Miguel Genebat, Ezequiel Ruiz-Mateos, Manuel Leal, Susana García-Obregón, Olatz Zenarruzabeitia, Francisco Borrego

**Affiliations:** ^1^Immunopathology Group, Biocruces-Bizkaia Health Research Institute, Barakaldo, Spain; ^2^Clinical Unit of Infectious Diseases, Microbiology and Preventive Medicine, Institute of Biomedicine of Seville (IBiS), Virgen del Rocío University Hospital, University of Seville, Consejo Superior de Investigaciones Científicas (CSIC), Sevilla, Spain; ^3^Internal Medicine Service, Santa Ángela de la Cruz Viamed Hospital, Sevilla, Spain; ^4^Pediatric Oncology Group, Biocruces-Bizkaia Health Research Institute, Barakaldo, Spain; ^5^Ikerbasque, Basque Foundation for Science, Bilbao, Spain; ^6^Basque Center for Transfusion and Human Tissues, Galdakao, Spain

**Keywords:** CD300, CD300a, human immunodeficiency virus-1, CD4 T cells, PD1

## Abstract

The ability of the CD300a inhibitory receptor to modulate immune cell functions and its involvement in the pathogenesis of many diseases has aroused a great interest in this molecule. Within human CD4+ T lymphocytes from healthy donors, the inhibitory receptor CD300a is differentially expressed among different T helper subsets. However, there are no data about the expression and regulation of CD300a receptor on CD4+ T cells from human immunodeficiency virus (HIV)-1-infected patients. The objective of this study was to investigate the expression of CD300a on CD4+ T cells from HIV-infected patients on suppressive combined antiretroviral therapy (cART) and cART naïve patients. Our results have demonstrated that the expression levels of this inhibitory receptor were higher on CD4+ T cells from HIV-1 infected subjects compared with healthy donors, and that cART did not reverse the altered expression of CD300a receptor in these patients. We have observed an increase of CD300a expression on both PD1+CD4+ and CD38+CD4+ T cells from HIV-1 infected people. Interestingly, a triple positive (CD300a+PD1+CD38+) subset was expanded in naïve HIV-1 infected patients, while it was very rare in healthy donors and patients on cART. Finally, we found a negative correlation of CD300a expression on CD4+ T lymphocytes and some markers associated with HIV-1 disease progression. Thus, our results show that HIV-1 infection has an impact in the regulation of CD300a inhibitory receptor expression levels, and further studies will shed light into the role of this cell surface receptor in the pathogenesis of HIV infection.

## Introduction

In order to mount an adequate and effective response against offenses, the immune system is orchestrated in part by a balance between stimulatory and inhibitory signals transmitted by a variety of receptors found on the surface of immune cells. Human CD300a is a member of the CD300 receptor family whose genes are located on chromosome 17 (1, 2). CD300a is a type I transmembrane protein formed by an immunoglobulin (Ig)V-like extracellular domain, a transmembrane region, and a long cytoplasmic tail containing three classical and one non-classical immunoreceptor tyrosine-based inhibitory motifs, which provides the receptor with an inhibitory capacity (1–5). Regarding to the ligands, CD300a is known to recognize phosphatidylserine (PS) and phosphatidylethanolamine (PE), phospholipids that are found in the inner leaflet of plasma membrane in resting cells and are exposed to the outer leaflet when cells get activated, transformed, infected, or dead (6–8).

Human CD300a receptor is found on the surface of both myeloid and lymphoid cells. Within human CD4+ T lymphocytes, it is differentially expressed among different subsets, and newborns CD4+ T cells significantly express lower levels of this receptor than adults (9, 10). Human Th1 cells expressing CD300a tend to be polyfunctional and after stimulation express the transcription factor eomesodermin (Eomes) (9). CD300a inhibitory receptor has the capacity to modulate CD4+ T cell responses through a signaling pathway that involves the src homology 2 domain containing protein tyrosine phosphatase (SHP)-1 (3, 11). Specifically, the co-ligation of the T cell receptor (TCR) and CD300a inhibited the calcium mobilization evoked by TCR alone and modulated the interferon (IFN)γ production on Th1 polarized cells (11). Importantly, over the past few years, several publications have emphasized the significant role that CD300a has in complex biological processes such as cytokine production and phagocytosis, and in a diversity of diseases including autoimmune disorders, allergic and inflammatory diseases, hematological malignancies, viral infections, etc. (2, 12–15).

Several studies have described the role of CD300a and its ligands PS and PE in the mechanisms that viruses use to enter host cells and also to evade the attack of the immune system. It is well known that one of the strategies used by viruses is apoptotic mimicry. Through this mechanism, which consists of enclosing viruses in a lipid bilayer obtained from the plasma membrane of host cells, they impersonate apoptotic cells and debris by concentrating PS and PE within their membranes, as it is the case of enveloped viruses; or by covering themselves in cell-derived PS and PE-containing vesicles, as it happens for many non-enveloped viruses (16–18). In this context, it has been shown that the interaction of CD300a with PS and PE enhances the infection of Dengue virus and other mosquito-borne viruses such as Yellow fever, West Nile, and Chikungunya viruses (14). In addition, Grauwet et al. have described that the US3 protein kinase of pseudorabies virus induces the exposure of PS and PE on the infected cells, a process that depends on the kinase activity of US3 and on group I p21-activated kinases. In consequence, the binding of CD300a on NK cells to the infected cells is increased, leading to an inhibition of NK cell-mediated killing (15). As a matter of fact, NK cells express high levels of CD300a (4, 5, 8, 10).

There are few reports describing the potential implication of CD300a during human immunodeficiency virus (HIV) infection. In a first study, a positive correlation between the mRNA levels of CD300a and the levels of basic leucine zipper transcription factor ATF-like (BATF) was described on HIV-specific CD8+ T cells. PD1, a marker of exhausted T cells, inhibits their function by upregulating BAFT, which has been suggested to increase other negative feedback pathways, including the expression of the CD300a inhibitory receptor, indicating that this molecule could also have a role in T cell exhaustion during HIV infection (19). Other report described a downregulation of CD300a expression on B cells from HIV-infected individuals, which could explain in part the B cell hyper-activation and dysfunction found in these patients (20). The downregulation in CD300a expression on B cell subsets was not corrected by effective antiretroviral therapy. On the other hand, a correlation between CD4+ T cell count and CD300a expression on memory B cells was observed in patients whose viremia was controlled by combined antiretroviral therapy (cART) (20). Finally, we have recently described the expression of CD300 receptors on monocytes from chronically HIV-1 infected patients under cART and its association with monocyte cytokine production. However, regarding to CD300a expression on monocytes, no differences were found between healthy donors and cART-treated HIV-1-infected patients (21).

In terms of CD4+ T cells, the expression of the CD300a receptor in HIV-infected patients and its regulation is still unknown. In this study, we have found that the cell surface levels of this inhibitory receptor are increased on CD4+ T cells after HIV-1 infection and it is not reverted by cART. Interestingly, a triple positive (CD300a+PD1+CD38+) subset was expanded in cART naïve HIV-1 infected patients, while it was very rare in healthy donors and cART-treated patients. We have also found an association between CD300a expression on CD4+ T lymphocytes and HIV disease progression markers.

## Materials and Methods

### Subjects and Samples

In this work, frozen peripheral blood mononuclear cells (PBMCs) from healthy adult donors and chronically HIV-1 infected patients were analyzed. Blood samples from healthy donors (*n* = 19) were obtained from the Basque Biobank for Research (http://www.biobancovasco.org). All subjects provided written and signed informed consent in accordance with the Declaration of Helsinki. The study was approved by the Basque Ethics Committee for Clinical Research (PI2014017 and PI2013108). Plasma and PBMC cryopreserved samples from asymptomatic HIV-1 infected patients on suppressive cART (viral load < 20 copies/mL) for at least 6 months (*n* = 16) and from cART naïve HIV-1 infected subjects (viral load > 9,300 copies/mL) (*n* = 20), were collected from the Virgen del Rocío University Hospital in Seville (Spain). Clinical data of cART-treated and cART naïve HIV-1 infected patients are described in Tables S1 and S2 in Supplementary Material, respectively. All patients provided written and signed informed consent in accordance with the Declaration of Helsinki. The study was approved by the Virgen del Rocío University Hospital Ethics Committee for Research (15/2009).

### Laboratory Methods

CD4 T-cell counts were determined in fresh whole blood using an Epic XL-MCL flow cytometer (Beckman-Coulter, Brea, CA, USA) according to the manufacturer’s instructions. Plasma HIV-1 RNA concentration was measured using quantitative polymerase chain reaction (COBAS Ampliprep/COBAS Taqman HIV-1 test, Roche Molecular Systems, Basel, Switzerland) according to the manufacturer’s protocol. The detection limit for this assay was 20 HIV RNA copies per milliliter.

High-sensitive C-reactive protein (hsCRP) and β2-microglobulin were determined with an immunoturbidimetric assay using COBAS 701 (Roche Diagnostics, GmbH, Mannheim, Germany). d-dimer levels were determined using an automated latex enhanced immunoassay (HemosIL, d-Dimer HS 500, Instrumentation Laboratory) in plasma samples stored at −20°C.

### Flow Cytometry Analysis

To perform flow cytometry-based procedures, the following fluorochrome-conjugated monoclonal mouse anti-human antibodies were used: PerCP-Cy5.5 anti-PD1 (clone EH12.1), PE-Cy7 anti-CD3 (clone SK7), APC anti-CD38 (clone HIT2) and BV421 anti-CD4 (clone RPA-T4) from BD Biosciences; PE anti-CD300a (clone E59.126) from Beckman Coulter; APC-eFluor780 anti-CD27 (clone O323) from eBioscience and BV510 anti-CD45RA (clone HI100) from BioLegend. Frozen PBMCs were thawed at 37°C and washed with PBS two times. Afterward, cells were stained with LIVE/DEAD Fixable Near-IR Dead Cell Stain Kit (Life Technologies) to test for viability, followed by a second step of incubation with different fluorochrome-conjugated antibodies to assess the expression of surface markers. In both steps, PBMCs were incubated for 30 min on ice protected from the light and were washed twice with 2.5% of bovine serum albumin in PBS. Finally, cells were fixed with 200 µl of 4% of paraformaldehyde (Sigma-Aldrich) for 15 min at 4°C and then other 200 µl of PBS were added. Samples were acquired with a flow cytometer FACS Canto II (BD Biosciences), using the FACS Diva software (BD Biosciences). Data were analyzed with FlowJo 10.0.7 software.

### Enzyme-Linked Immunosorbent Assay (ELISA)

Enzyme-Linked Immunosorbent Assays were performed to measure soluble markers in the plasma samples from HIV-1-infected patients: sCD163 (Macro163™, IQ Products) and sCD14 (Human CD14 ELISA Kit, Diaclone Immunology Products) levels were determined. ELISA experiments were performed following the manufacturers’ protocol.

### Data Representation and Statistical Analysis

GraphPad Prism software (version 6.01) was employed for the graphical representation and statistical analysis. Data were represented in scatter dot plots with the median or bar graphs showing the mean with SEM. Prior to statistical analyses, data were tested for normal distribution with D’Agostino and Pearson normality test. Then, parametric tests were applied when the normality test was passed, while non-parametric tests were utilized when it was not. Differences between healthy donors and naïve and cART-treated HIV-1 infected patients were evaluated with the non-parametric unpaired Mann–Whitney test, and the comparisons within cell subsets from each subject were made with the non-parametric Wilcoxon matched-pairs test. The same software was used to carry out correlation analysis. In this case, parametric Pearson correlation test or non-parametric Spearman test was employed depending on data distribution. Boolean gate analysis were done utilizing the FlowJo 10.0.7 software and data were represented in pie chart graphs using SPICE v5.3 software and bar graphs showing the mean with SEM utilizing GraphPad Prism software.

## Results

### CD300a Expression on CD4+ T Lymphocytes From HIV-Infected Patients

**Figure 1:**
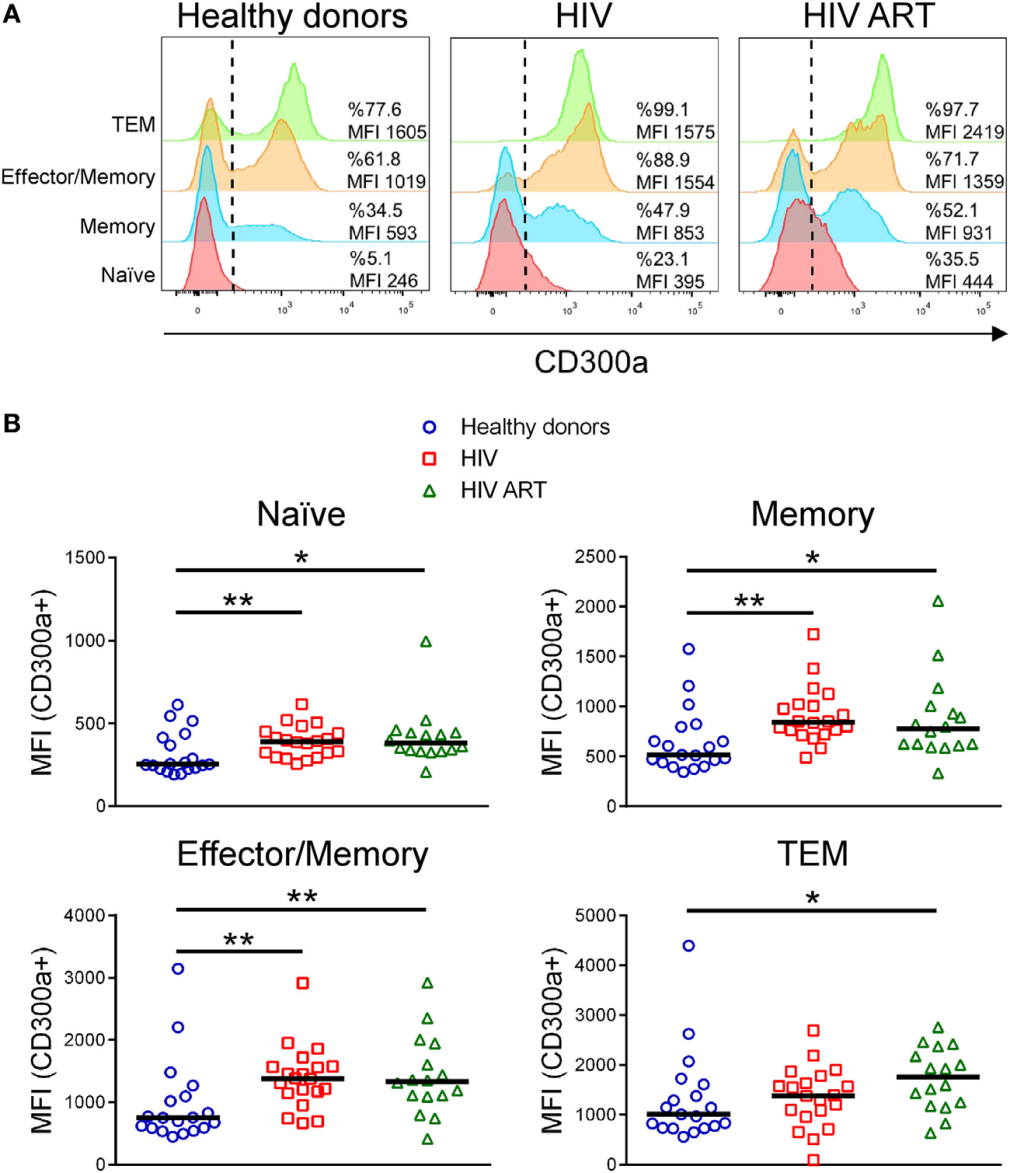
CD300a inhibitory receptor expression on CD4+ T cell subsets from healthy donors and human immunodeficiency virus (HIV)-1 infected patients. **(A)** Representative histograms showing the percentage of CD300a+ cells and the median fluorescence intensity (MFI) of CD300a within positive cells on CD4+ T cell subpopulations. Data from a representative healthy donor, a cART naïve (HIV) and a patient on cART (HIV ART) are shown. **(B)** Dot plots showing the MFI of CD300a within positive cells on CD4+ T cell subsets from healthy donors and cART naïve (HIV) and cART-treated (HIV ART) HIV-1-infected patients. Each dot represents a subject and the median is shown. *p < 0.05, **p < 0.01.

The human CD300a inhibitory receptor is known to be differentially expressed on CD4+ T cell subpopulations (9–11). Here, we have analyzed the CD300a expression levels on CD4+ T cell subsets from HIV-1 infected patients in comparison with healthy donors. CD27 and CD45RA cell surface receptors were used as markers to identify different CD4+ T subpopulations: naïve (CD27+CD45RA+), memory (CD27+CD45RA−), effector/memory (CD27−CD45RA−), and terminal differentiated effector/memory (TEM) (CD27−CD45RA+) cells (Figure S1A in Supplementary Material). In agreement with previous publications, the frequency of each subset varied between the different groups of patients (Figure S1B in Supplementary Material) (22–24). First, we examined the expression of CD300a on CD4+ T cell subsets from healthy donors, cART naïve, and patients on cART. Similar to other published findings (10), we have found that in healthy donors, naïve CD4+ T cells exhibited the lowest CD300a expression levels while TEM cells displayed the highest. Naïve cells were negative or expressed low levels of CD300a and almost all TEM cells were positive and expressed high levels of CD300a (Figure 1A). On the other hand, memory and effector/memory CD4+ T cells are divided into two well defined CD300a+ and CD300a− subpopulations (Figure 1A). Regarding CD4+ T cells from HIV-1 infected patients, we observed a similar CD300a expression pattern among the four subpopulations (Figure 1A).

Next, we compared the percentage of CD300a+ cells on CD4+ T cells from healthy donors with the cells from cART naïve and cART-treated HIV-1 infected patients. In general, the results did not show significant differences between groups (Figure S2 in Supplementary Material). In addition, we also checked the median fluorescence intensity (MFI) of CD300a within the CD300a+ cells in the same CD4+ T cell subpopulations, as a measure of the amount of CD300a molecules on the surface of CD300a+ cells. We observed a significant upregulation of CD300a expression on CD4+ T cells from HIV-1-infected patients when compared with cells from healthy donors. This was observed in every CD4+ T cell subset, with the exception of TEM cells, which only exhibited significant differences between healthy donors and cART-treated patients (Figure 1B). Therefore, our results demonstrated that HIV-1 infection increases CD300a expression levels on CD4+CD300a+ cells and that cART does not reverse the upregulation of CD300a expression found in these patients.

### CD300a Expression on PD1+CD4+ T Cells

During chronic HIV-1 infection, the persistent antigen exposure gives rise to T cell exhaustion, which is characterized by a reversible loss of effector functions and proliferative capacity. Exhausted T cells are characterized by a higher expression of inhibitory receptors such as PD1, TIM-3, and LAG-3 (25, 26). The PD1 inhibitory receptor, inductively expressed on T cells upon activation, has been identified as a major regulator of T cell exhaustion during chronic HIV infection (19, 27, 28). We decided to measure the expression of CD300a receptor on PD1+CD4+ T lymphocytes from HIV-1 infected patients. First, we were able to reproduce the results obtained in previous works (22, 24), since we observed an increase in the percentage of PD1+ cells on memory, effector/memory and TEM CD4+ T cells from HIV-viremic patients that are not receiving cART (Figure S3 in Supplementary Material). The frequency of PD1+ cells was significantly diminished with cART on the same CD4+ T cell subsets from HIV-1-infected patients (Figure S3A in Supplementary Material).

**Figure 2:**
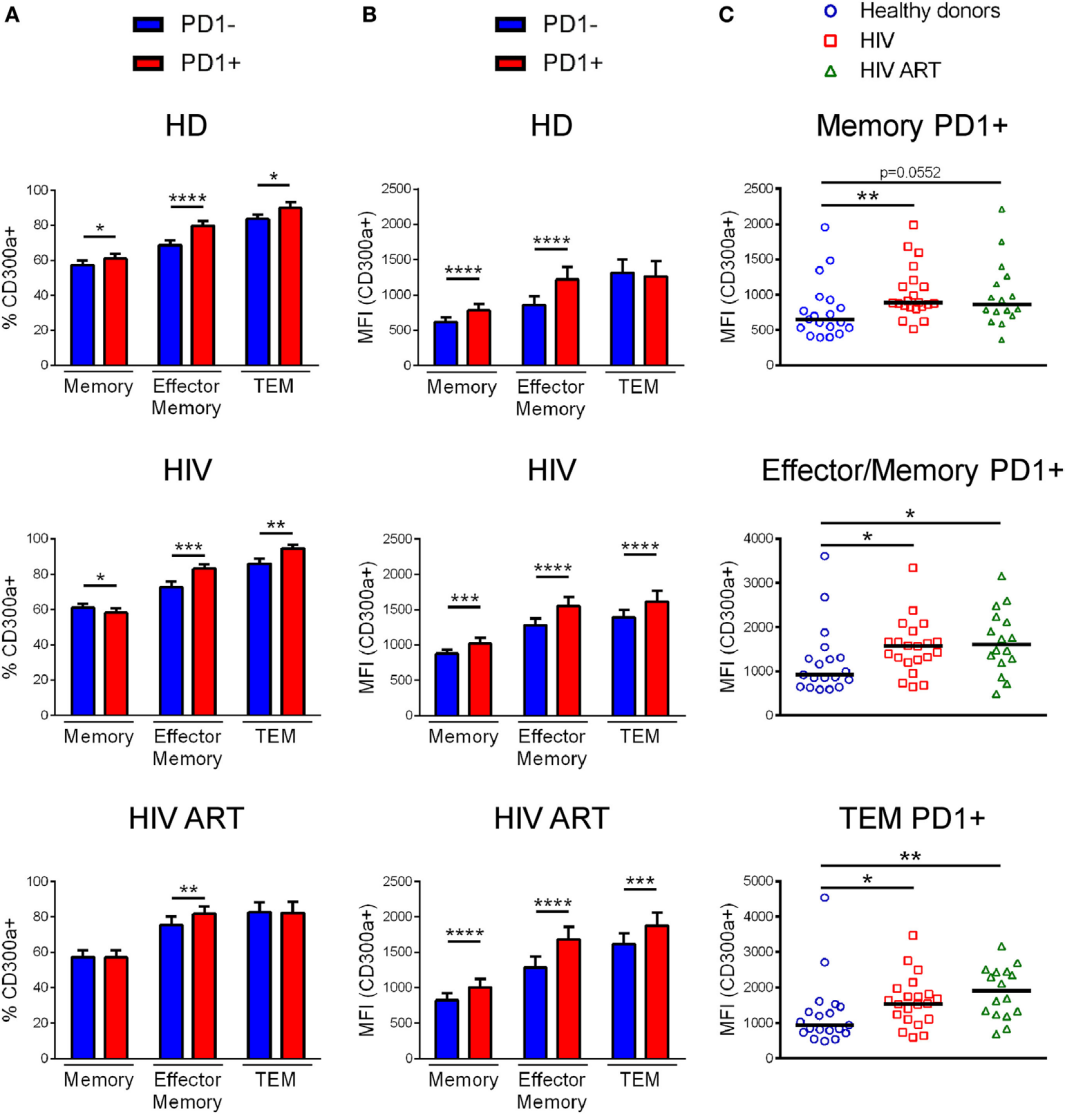
CD300a expression on PD1+CD4+ T lymphocytes. Bar graphs showing the percentage of CD300a+ cells **(A)** and the median fluorescence intensity (MFI) of CD300a within positive cells **(B)** in PD1+CD4+ and PD1-CD4+ T lymphocytes from healthy donors, cART naïve [human immunodeficiency virus (HIV)] and patients on cART (HIV ART). Error bars represent the SEM. **(C)** Dot plots representing the MFI of CD300a within CD4+ T cells expressing both CD300a and PD1 from healthy donors, cART naïve (HIV) and cART-treated (HIV ART) HIV-1 infected patients. Each dot represents a subject and the median is shown. *p < 0.05, **p < 0.01, ***p < 0.001, ****p < 0.0001.

Afterward, we analyzed the percentage of CD300a+ cells within PD1+ and PD1− CD4+ T lymphocytes from healthy donors, cART naïve, and cART-treated HIV-1 infected patients (Figure S3B in Supplementary Material). Specifically, we focused on memory, effector/memory and TEM CD4+ T cells. Naïve CD4+ T cells were not analyzed since they express negligible levels of PD1, as we (Figure S3A in Supplementary Material) and others have shown (24, 29). Regarding to effector/memory and TEM CD4+ T cell subsets we saw that PD1+ cells exhibited a higher percentage of CD300a+ cells than PD1− cells, in both healthy donors and HIV-1 infected patients with the exception of TEM cells from cART-treated patients, which did not display any difference between PD1+ and PD1− cells (Figure 2A). On the other hand, the frequency of CD300a+ cells in the PD1+ and PD1− memory CD4+ T lymphocytes were somehow different between the groups of patients (Figure 2A). Importantly, when we measured the MFI of the CD300a on PD1+CD300a+ and PD1−CD300a+ cells, we observed that PD1+ cells in all CD4+ T cell subsets always exhibited higher levels of expression of CD300a than PD1− cells (Figure 2B).

Finally, we compared the MFI of CD300a on PD1+CD300a+ cells from healthy donors with the cells from HIV-1 infected patients. We observed that PD1+ cells from both cART naïve and cART-treated HIV-1-infected patients displayed a higher MFI of CD300a than cells from healthy donors (Figure 2C). Altogether, these results indicate that HIV-1 infection induces an increase on the expression of CD300a inhibitory receptor on CD4+ T cells that otherwise already express the checkpoint PD-1.

### CD300a Expression on CD38+CD4+ T Cells

Human immunodeficiency virus-1 infection induces T cell (CD4+ and CD8+) dysfunction and activation, which consequently upregulates the expression of the surface receptor CD38 on these cells. In fact, high expression of CD38 on T lymphocytes is known to predict HIV infection progression (30–32). Therefore, we decided to utilize the marker CD38 in order to investigate the expression of CD300a receptor on activated CD4+ T cells from HIV-1 infected people. In agreement with previous publications (30, 33), we observed that, except for the naïve subset, all CD4+ T cells from cART naïve HIV-1 infected patients express higher percentages of CD38+ cells than the cells from healthy donors or cART-treated patients (Figure S4A in Supplementary Material). There were no significant differences in the frequency of CD38+ cells on naïve CD4+ T cells from healthy subjects and infected patients (Figure S4A in Supplementary Material). In fact, resting naïve CD4+ T cells express constitutively low levels (MFI) of CD38, making this receptor a proper activation marker only for T cells with a non-naïve phenotype (30). Hence, naïve CD4+ T cells were not considered in the following analysis.

**Figure 3:**
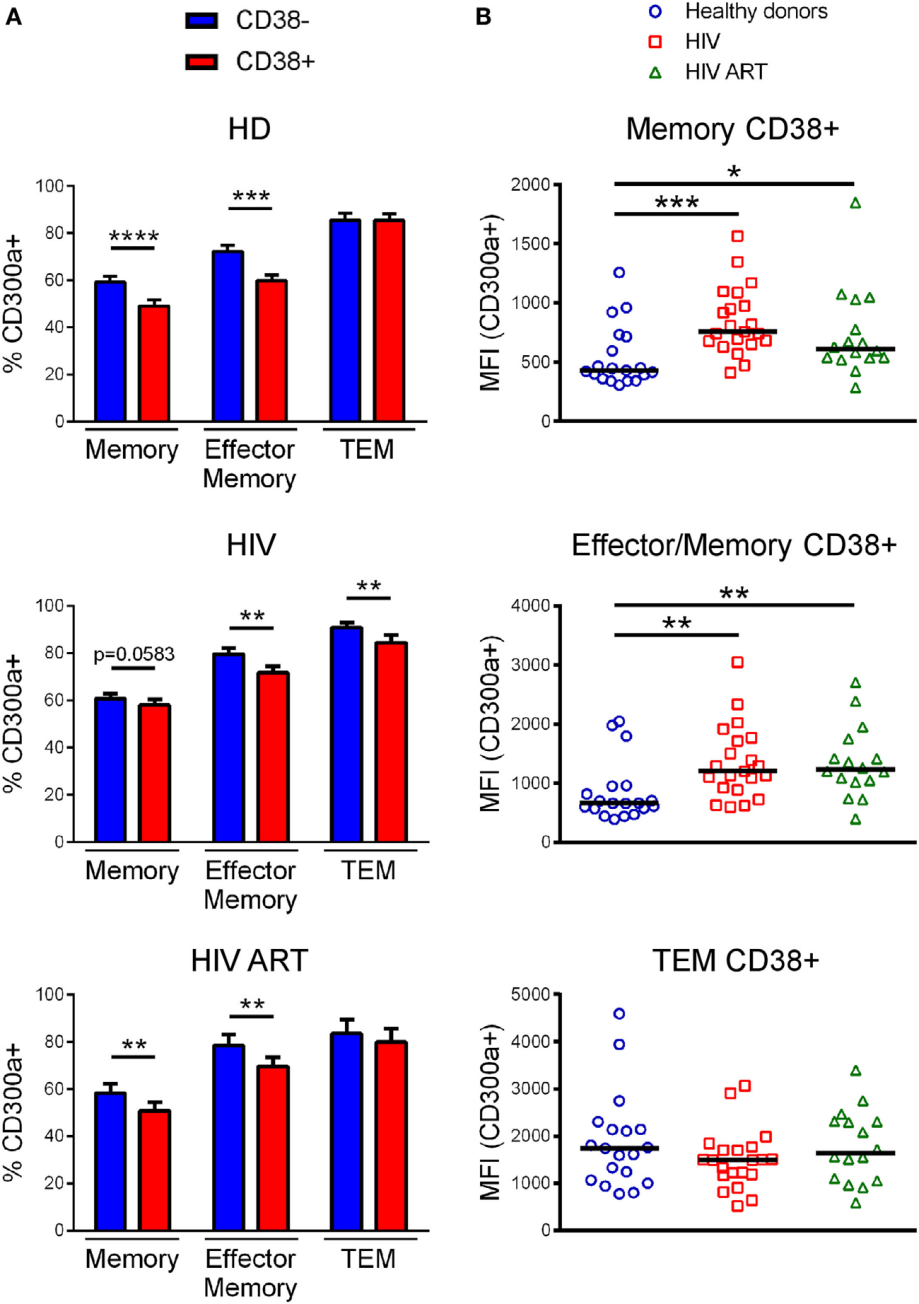
CD300a expression on CD38+CD4+ T lymphocytes. **(A)** Bar graphs representing the percentage of CD300a+ cells within CD38+CD4+ and CD38−CD4+ T lymphocytes from healthy donors, cART naïve [human immunodeficiency virus (HIV)] and patients on cART (HIV ART). Error bars represent the SEM. **(B)** Dot plots representing the median fluorescence intensity of CD300a within CD4+ T cells expressing both CD300a and CD38 from healthy donors, cART naïve (HIV), and patients on cART (HIV ART). Each dot represents a subject and the median is shown. *p < 0.05, **p < 0.01, ***p < 0.001, ****p < 0.0001.

We determined the percentage of CD300a+ cells on CD38+CD4+ T lymphocytes, focusing on memory, effector/memory, and TEM CD4+ T cells, from both healthy donors and infected patients (Figure S4B in Supplementary Material). In all subjects, memory and effector/memory CD4+ T cells expressing CD38 exhibited a lower percentage of CD300a+ cells than CD38− cells (Figure 3A). The same result was obtained from TEM cells but only in cART naïve patients (Figure 3A). Importantly, when we compared the MFI of CD300a on CD38+CD300a+ cells from healthy donors with the cells from cART naïve and cART-treated patients, we saw a higher expression of the CD300a receptor on memory and effector/memory CD4+ T cells from HIV-1-infected patients (Figure 3B).

**Figure 4:**
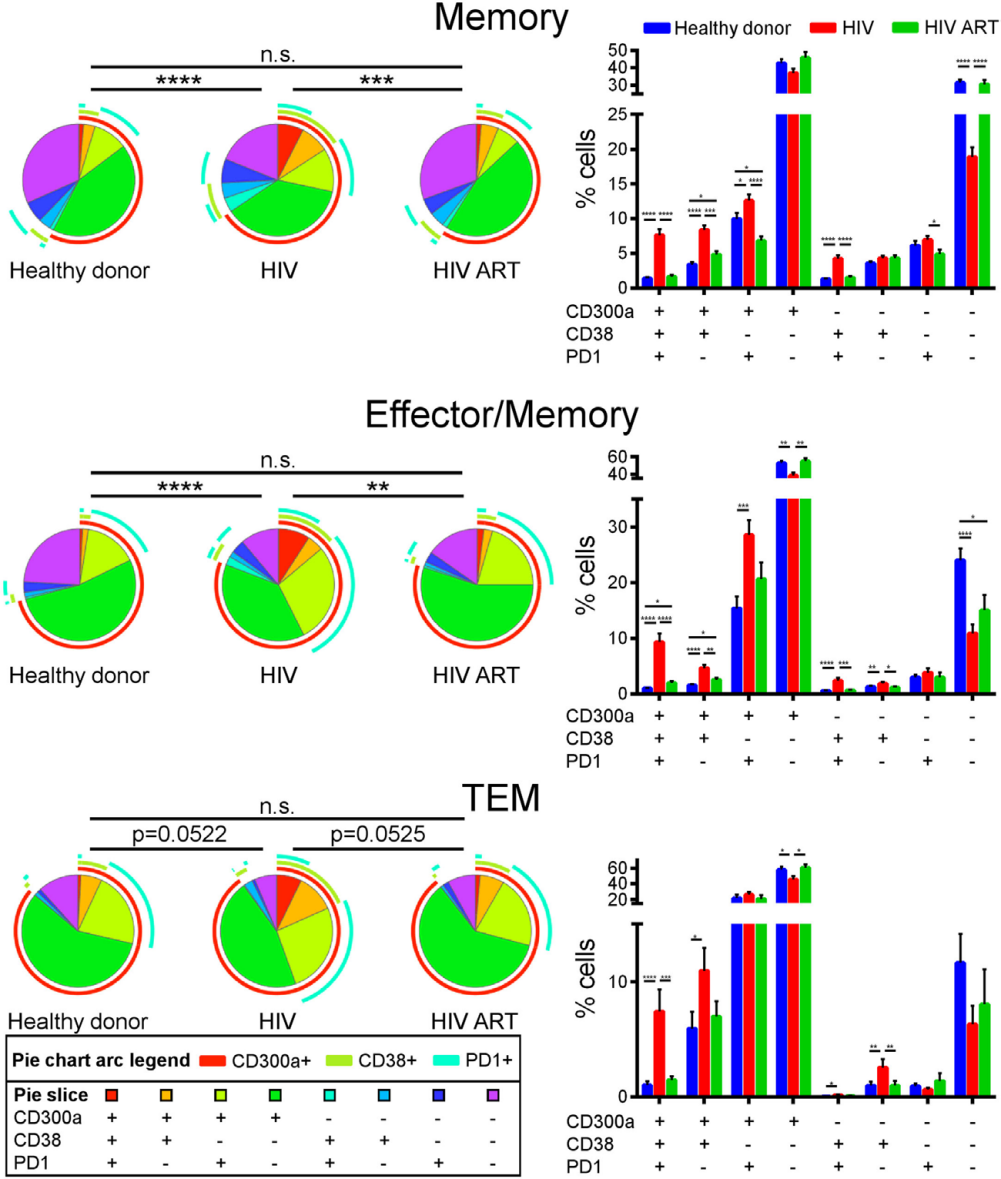
Co-expression of CD300a, PD1, and CD38 on CD4+ T cells. Boolean gate analysis showing the frequency of memory, effector/memory, and TEM CD4+ T cells expressing CD300a, PD1, and CD38, in healthy donors, cART naïve [human immunodeficiency virus (HIV)], and patients on cART (HIV ART). Data are represented in pie chart graphs and bar graphs showing the mean with SEM. *p < 0.05, **p < 0.01, ***p < 0.001, ****p < 0.0001.

We finally carried out a Boolean gate analysis, in order to compare the co-expression of CD300a, PD1, and CD38 receptors on non-naïve CD4+ T cells from healthy donors and cART naïve and cART-treated HIV-1 infected patients. Regarding memory and effector/memory CD4+ T lymphocytes, we saw a higher percentage of triple positive (CD300a+PD1+CD38+) and double positive (CD300a+PD1+CD38−, CD300a+PD1−CD38+ and CD300a−PD1+CD38+) cells in cART naïve patients than in healthy donors or cART-treated patients. The percentage of triple negative cells (CD300a−PD1−CD38−) was lower in cART naïve patients. TEM CD4+ T cells displayed similar results, mainly in the frequency of triple-positive cells (Figure 4). Altogether, we observed that in terms of CD300a, PD1, and CD38 expression, CD4+ T cells from healthy donors and cART-treated patients were very similar, while the CD4+ cells from cART naïve HIV-1 infected patients exhibited a clearly different phenotype with a very interesting appearance of a significant triple positive (CD300a+PD1+CD38+) cell subset (Figure 4).

### Association of CD300a Expression With Markers of HIV-1 Infection Progression

**Figure 5:**
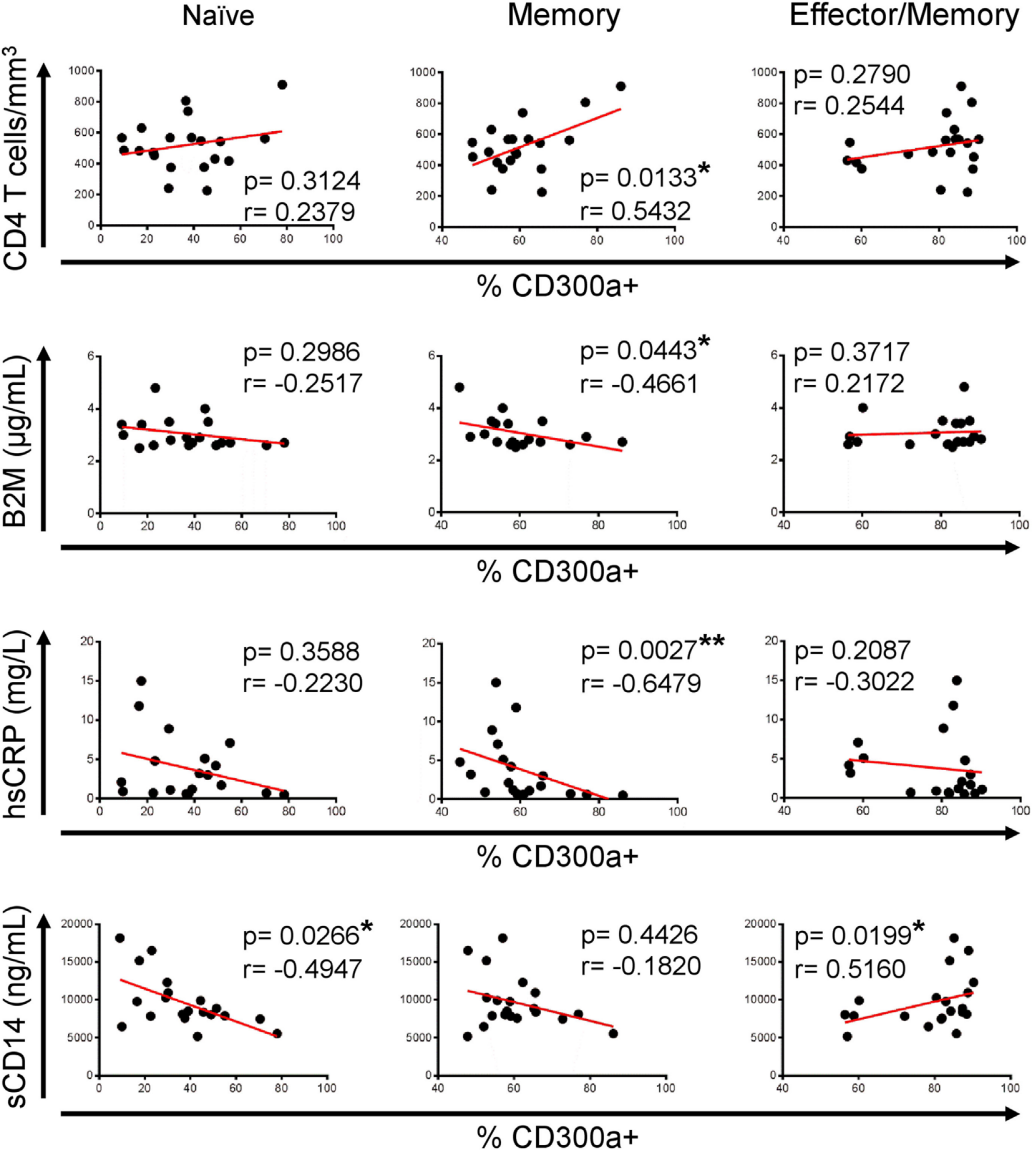
Association of CD300a expression on CD4+ T lymphocytes with markers of human immunodeficiency virus (HIV)-1 infection progression in cART naïve HIV-1 infected patients. Correlation analysis of the percentage of CD300a+ cells on naïve (left column), memory (middle column), and effector/memory (right column) CD4+ T lymphocytes, with CD4+ T cell counts and β2-microglobulin (B2M), high sensitive C-reactive protein (hsCRP), and soluble CD14 (sCD14) levels in cART naïve HIV-1 infected patients. *p < 0.05, **p < 0.01.

Once we have described that CD300a expression is upregulated on CD4+ T lymphocytes from HIV-1 infected patients, we investigated if these findings could have any clinical relevance to determine HIV-1 disease progression. To do that, correlation analysis were carried out between CD300a expression and markers that are currently used for clinical monitoring of HIV-1 infected patients, including viral load and CD4+ T cell count, and also several soluble markers from plasma such as β2-microglobulin, d-dimer, hsCRP, soluble CD163 (sCD163), and soluble CD14 (sCD14). Figure S5 in Supplementary Material shows the values of each soluble marker and CD4+ T cell counts in both cohorts of patients. As expected, HIV-1-infected patients under cART exhibited a higher CD4+ T cell count than non-treated patients. In addition, cART-treated patients had lower serum levels of β2-microglobulin and sCD163 than cART naïve patients (Figure S5 in Supplementary Material). No correlation was found between the percentage of CD300a+ cells and viral load or d-dimer and sCD163 levels (data not shown). On the other hand, we did observe a significant correlation between CD4+ T cell counts, β2-microglobulin, hsCRP, and sCD14 levels and the frequency of CD300a+ cells in untreated HIV-1 infected patients. The percentage of CD300a+ cells within memory CD4+ T lymphocytes was positively associated with CD4+ T cell count, while it was negatively correlated with β2-microglobulin and hsCRP levels (Figure 5). Moreover, naïve CD4+ T cells from cART naïve patients also exhibited a negative association between the percentage of CD300a+ cells and sCD14 plasma levels, and the same tendency was observed on memory CD4+ T cells (Figure 5). In contrast, related to the effector/memory CD4+ T cells, we observed a somehow contradictory correlation pattern of the frequency of CD300a+ cells with the progression markers, as for example, the positive correlation between the percentage of CD300a+ cells and sCD14 levels (Figure 5), making it difficult to obtain a clear interpretation of the results in this CD4+ T cell subset from untreated patients.

**Figure 6:**
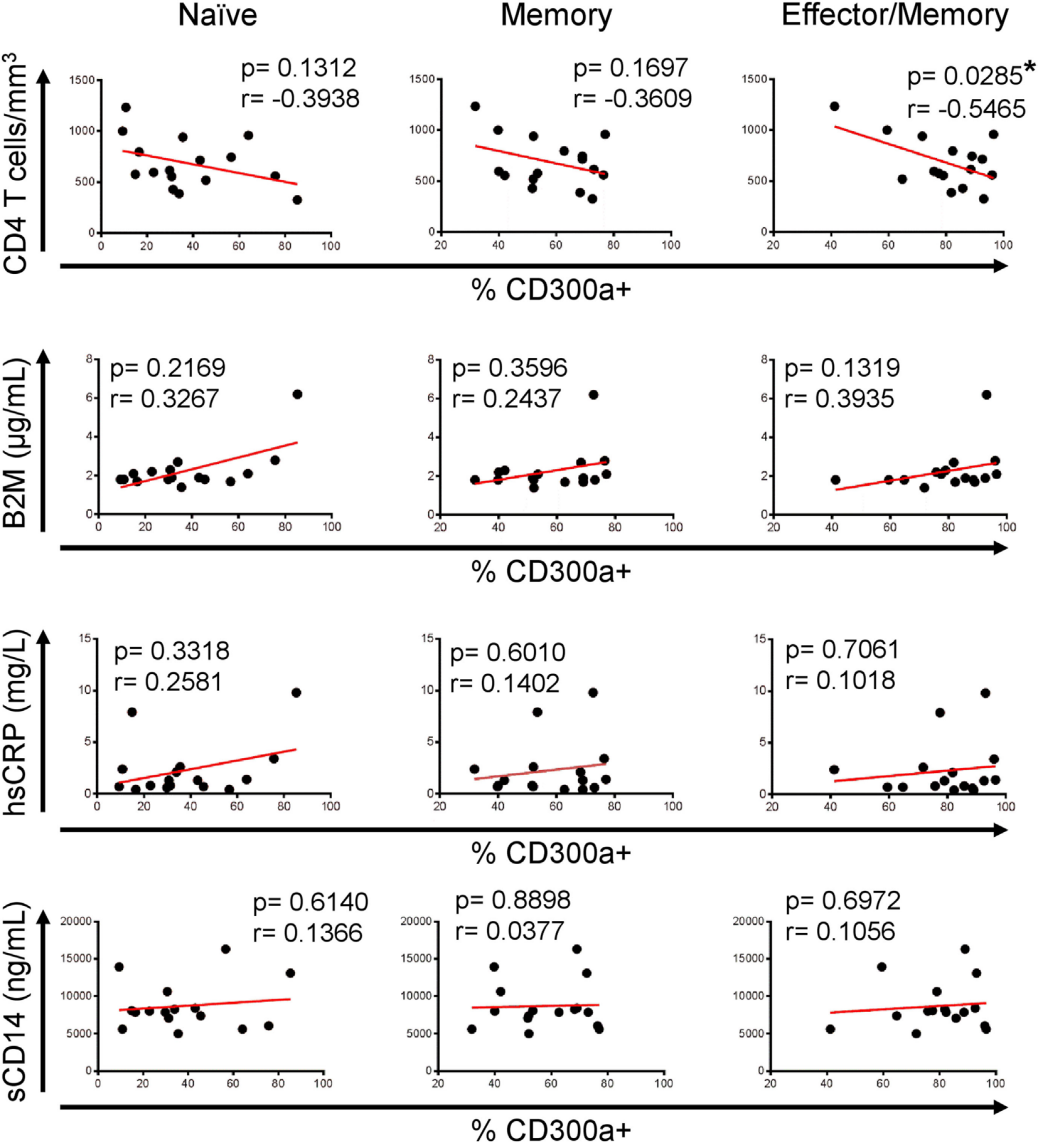
Association of CD300a expression on CD4+ T lymphocytes with markers of human immunodeficiency virus (HIV)-1 infection progression in HIV-1 infected patients on cART. Correlation analysis of the percentage of CD300a+ cells on naïve (left column), memory (middle column), and effector/memory (right column) CD4+ T lymphocytes, with CD4+ T cell counts and β2-microglobulin (B2M), high sensitive C-reactive protein (hsCRP), and soluble CD14 (sCD14) levels in HIV-1-infected patients on cART. *p < 0.05.

Interestingly, and in opposition to the analyses performed with samples from untreated patients, regarding naïve or memory CD4+ T cells from cART-treated patients, we did not see any correlation between the studied parameters, but an opposite tendency was noticed (Figure 6). Furthermore, effector/memory cells from HIV ART displayed a significant negative correlation between the percentage of CD300a+ cells and CD4+ T cell numbers (Figure 6). TEM CD4+ T cells from both untreated and cART-treated HIV-1 infected patients did not show any significant correlation (data not shown) with the CD4+ T cell count and serum levels of soluble markers. Altogether, these results suggest that a higher frequency of CD300a+ cells on CD4+ T cells could be associated to a better progression in cART naïve HIV-1-infected people, while this association disappears after the introduction of cART.

## Discussion

The ability of the CD300a inhibitory receptor to modulate immune cell effector functions and its involvement in the pathogenesis of several diseases has recently triggered a great interest in this molecule (1, 2, 12, 13). The CD300a receptor is known to have an important role in various viral infections, enhancing viral entry and promoting viral escape from the immune system (14, 15, 34). However, there are only a few publications about CD300a on HIV-1 infection. These publications indicate a possible role for CD300a in the exhaustion of HIV-specific CD8+ T cells (19) and in the B cell hyper-activation and dysfunction found in HIV-1 infected patients (20). In this work, we report that the expression levels of the CD300a inhibitory receptor are increased on CD4+ T cells from HIV-1-infected patients. Moreover, we found a CD4+ T cell population co-expressing PD1, CD38, and CD300a that is expanded in cART naïve HIV-1 infected patients, while in healthy donors and cART-treated patients is rare. We also discovered an association of CD300a expression with some markers of disease progression in non-treated HIV-1-infected patients.

The differential expression of CD300a inhibitory receptor among CD4+ T cell subpopulations was previously described in healthy donors (9–11). Here, we first observed that the expression pattern in the CD4+ T cell subsets in HIV-1 infected patients was similar to the one exhibited by healthy donors: naïve CD4+ T cells displayed the lowest levels of CD300a while TEM cells the highest and memory and effector/memory CD4+ T cells expressed intermediate levels. But importantly, we have discovered that the expression levels of the CD300a receptor in HIV-1 infected patients were significantly altered when we compared them with the expression on CD4+ T cells from healthy donors. Hassouneh et al. have described a higher percentage of CD300a+ cells on total CD4+ T cells in healthy donors, mainly in middle-age and old people, which are seropositive for cytomegalovirus (CMV) (35). Published data have shown that there is a high incidence of CMV positive blood donors in Spain (36) and also of CMV positive organ donors (see report from the Spanish National Transplant Organization at www.ont.es/infesp/Memorias/Memoria%20Donación%202017.pdf). On the other hand, it is also well known that a high percentage of HIV-1 infected people are co-infected with CMV (50–80%) (37, 38). Our results showed no significant differences in the frequency of CD300a+ cells on total CD4+ T cells and in the different cell subsets (Figure S2 in Supplementary Material), which somehow could be explained by the high incidence of CMV in both healthy donors and HIV-1-infected patients. On the other hand, we observed a significant upregulation of CD300a MFI on CD4+CD300a+ T cells from HIV-1-infected patients (Figure 1). Future studies are required to determine the role of CMV infection on the altered expression of CD300a observed in HIV-1-infected patients.

The expression of CD300a molecule is regulated by different stimuli. For instance, CD300a expression is upregulated after TLR9 stimulation on memory B cells (20) and its expression also increases on monocytes stimulated with IFNγ and cultured under hypoxia conditions (39, 40). The reason of why the CD300a receptor is upregulated on HIV-1-infected CD4+ T cells is still unknown. Though, as other immunomodulatory receptors such as PD1, the CD300a inhibitory receptor may be inductively expressed after T cell activation as a regulatory mechanism during HIV-1 infection, and probably the cytokine environment has an important role determining the expression levels of CD300a. It has been previously published that Th1 polarization in the presence of IL-12 induces the generation of mostly CD300a+ cells in healthy donors (9). Moreover, patients with HIV infection display an increased IL-12 production *in vivo* and *ex vivo* (41). Thus, it could be possible that the higher IL-12 production, among others, during acute/early HIV infection, may induce the upregulation of CD300a and this overexpression might be maintained during chronic HIV infection. Clearly, more studies are required to investigate the factors leading to an increase in the expression levels of CD300a during HIV infection. On other hand, our results did not show significant differences in CD300a expression levels on CD4+ T cells between cART naïve and cART-treated HIV-1 infected people, meaning that cART does not reverse the upregulation of CD300a found in infected patients. This is in line with previous results published by us where the altered levels of CD300a expression on B cells are not reversed by cART (20). The maintenance of the higher expression levels of CD300a inhibitory receptor in cART-treated HIV-1-infected subjects could be a reflection of the continuous immune activation in these patients, even after cART. It is well known that although cART decreases viral load to undetectable levels, as HIV is not completely eradicated, the activation of the immune system still occurs (32, 42–45).

Consistent with the results described by Quigley et al., who showed a positive correlation between CD300a mRNA levels and BATF, a transcription factor downstream of PD1 that increases inhibitory pathways on HIV-specific exhausted CD8+ T cells (19), here, we have discovered a higher frequency of CD300a+ cells on PD1+ cells in comparison with PD1− cells within most of CD4+ T cell subsets from both healthy donors and HIV-1 infected patients. It is well known that PD1 is an inhibitory receptor that is upregulated after T cell activation as a negative feedback mechanism (27–29). Several publications have proposed that PD1, apart from inducing immune exhaustion, identify a particular T cell differentiation stage and effector function (46–48). For instance, memory PD1+CD4+ T lymphocytes from healthy donors and HIV-1 infected children preferentially secreted IFNγ and IL-17A (49). Previously, it has been described that in healthy donors, CD4+ T cells expressing CD300a were higher producers of IFNγ than CD300a− cells, and that they were more polyfunctional (9, 11). Therefore, CD300a receptor, as PD1, may represent a CD4+ T cell subset with specific effector functions, at least in healthy donors. But even more relevant for this study, the expression levels of the CD300a inhibitory receptor were significantly higher on PD1+CD4+ T lymphocytes from HIV-1-infected patients when compared with the same cells from healthy donors.

It is well known that HIV-1 induces T cell activation and consequently increases the expression of CD38 (30, 50). A higher CD38 expression on CD4+ T cells from viremic HIV-1-infected people is a biomarker of poor prognosis and is strongly associated with short survival in patients with advanced infection (30–32, 51). In this study, we saw a decrease in the percentage of CD300a+ cells within CD38+CD4+ T lymphocytes from both healthy people and HIV-1 infected patients, in comparison with CD38−CD4+ T cells. But importantly, CD38+ cells from HIV-1 infected patients exhibited higher expression levels of CD300a than the CD38+ cells from healthy donors, which is consistent with a general upregulation of CD300a expression levels on different CD4+ T cell populations after HIV-1 infection, regardless of the exhaustion or activation status of the cells. Finally, Boolean gate analysis showed that in terms of CD300a, PD1, and CD38 expression pattern, the phenotype of CD4+ T cells from healthy donors was very similar to the one of cART-treated HIV-1 infected people, while naïve patients for cART exhibited a different pattern. It is very possible that the differences found between cART-treated and non-treated HIV-1 infected subjects are mainly due to their disparities in PD1 and CD38 expression. Of note, these analyses revealed an expansion of a triple positive cell subset (CD300a+PD1+CD38+) in untreated patients that is very rare in healthy donors and HIV-1 patients on cART. The increased percentage of this triple positive population is very possibly the consequence of an increase in PD1+ and CD38+ cells in cART naïve patients rather than an increase in the frequency of CD300a+ cells. This subset may represent a highly activated population characterized by high expression of immune checkpoints. In the future, it will be very interesting to study in depth this subpopulation, both phenotypically (immune checkpoints) and functionally (proliferation, cytokine production, etc.).

Finally, we investigated if there was any association between CD300a expression and markers of HIV-1 disease progression. An increase in CD4+ T cell counts has been broadly interpreted as good prognosis in HIV-1-infected people (31, 52). Moreover, there are various soluble molecules that have been related to HIV-1 infection progression. Among others, high β2-microglobulin and hsCRP values in plasma reflect the activation of immune system (53, 54) and illustrate disease progression (54, 55). Furthermore, high plasma levels of sCD14, a marker of microbial translocation, have also been related to disease progression and poor response to cART in HIV-infected patients (56–58). Our results showed that in untreated HIV-1-infected patients, the percentage of cells expressing CD300a was positively correlated with CD4+ T cell numbers, while was negatively associated to β2-microglobulin, hsCRP, and sCD14 levels, suggesting that a higher frequency of CD300a+ cells could be indicative of good prognosis in active HIV-1 infection. In contrast, our results did not show the same tendency regarding to CD4+ T cells from HIV-infected patients receiving cART. In fact, an opposite pattern between naïve cART and treated patients was found. This could suggest that this receptor might play different roles depending on the levels of virus replication and T cell activation. Since T cell activation supports viral replication, in untreated HIV-1 patients, a higher expression of the CD300a inhibitory receptor might be beneficial. However in patients under effective cART, because viral levels decrease and consequently permit the antiviral effects to prevail, higher expression levels of CD300a inhibitory receptor could be counterproductive for T cell activation. Undoubtedly, further studies are required to test this hypothesis.

In summary, our study demonstrates that chronically HIV-1 infected patients exhibit an altered expression of CD300a inhibitory receptor on CD4+ T lymphocytes that is not reverted by effective cART. Moreover, we have discovered a negative correlation between CD300a levels and some markers of HIV-1 infection progression in cART naïve HIV-1 infected patients. These are promising findings that will lead to new research aimed at further understanding the role of the CD300a inhibitory receptor in CD4+ T lymphocytes during chronic HIV-1 infection.

## Ethics Statement

Institutional and Ethical Review Board approvals were obtained and written informed consent was obtained from all healthy donors and patients. The study was approved by the Basque Ethics Committee for Clinical Research and Virgen del Rocío University Hospital Ethics Committee for Biomedical Research.

## Author Contributions

JV designed and performed experiments, analyzed and interpreted the data, designed the figures, and wrote the manuscript. IT analyzed the results and made the figures. LG-U analyzed the results. AO participated in the interpretation of the data. ML and MG clinically characterized the patients and participated in the interpretation of the data. LT-D and ER-M collected samples, performed experiments, and analyzed the data. SG-O analyzed the results and participated in the interpretation of the data. OZ participated in the design of the study and interpreted the data. FB conceived and designed the study, interpreted the data, and wrote the manuscript. All the authors critically reviewed, edited, and approved the final manuscript.

## Conflict of Interest Statement

The authors declare that the research was conducted in the absence of any commercial or financial relationships that could be construed as a potential conflict of interest.
