# Supplementary material for: Altered Expression of CD300a Inhibitory Receptor on CD4+ T Cells From Human Immunodeficiency Virus-1-Infected Patients: Association With Disease Progression Markers
Source: Front Immunol. 2018 Jul 23;9:1709. doi: 10.3389/fimmu.2018.01709 (PMC6065254; doi:10.3389/fimmu.2018.01709)
Supplement: Supplementary file 1 [file Data_Sheet_1.PDF]

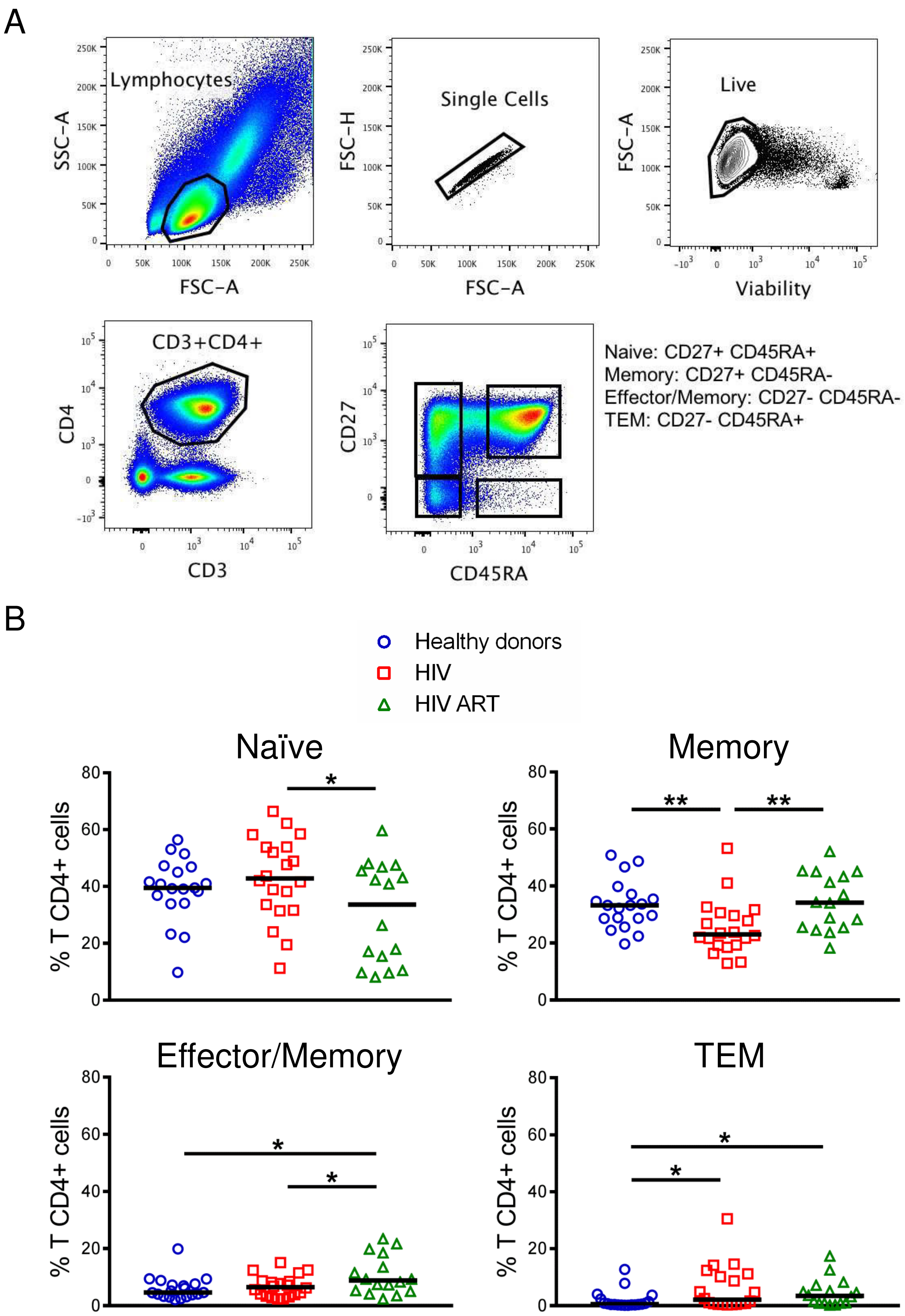
A
Lymphocytes
SSC-A
FSC-A
Single Cells
FSC-H
Live
Viability
CD3+CD4+
CD4
CD3
CD27
CD45RA
Naive: CD27+ CD45RA+
Memory: CD27+ CD45RA-
Effector/Memory: CD27- CD45RA-
TEM: CD27- CD45RA+
B
Healthy donors
HIV
HIV ART
Naïve
Memory
Effector/Memory
TEM
% T CD4+ cells

Supplementary Figure 1. CD4+ T cell subsets from healthy donors and HIV-1 infected patients. (A) Pseudocolor plots representing the gating strategy utilized during the study, data from a representative healthy donor is shown. Lymphocytes were electronically gated based on their forward and side scatter parameters, then live cells were selected and CD4+ T lymphocytes were detected by the expression of both CD3 and CD4. Four CD4+ T subsets were differentiated based on the expression of CD27 and CD45RA: naïve (CD27+CD45RA+), memory (CD27+CD45RA-), effector/memory (CD27-CD45RA-) and terminal differentiated effector/memory (TEM) (CD27-CD45RA+) cells. (B) Dot plot graphs showing the percentage of each CD4+ T cell subpopulation from healthy donors, cART naïve (HIV) and patients on cART (HIV ART). Each dot represents a subject and the median is shown. *$p<0.05$, ** $p<0.01$.

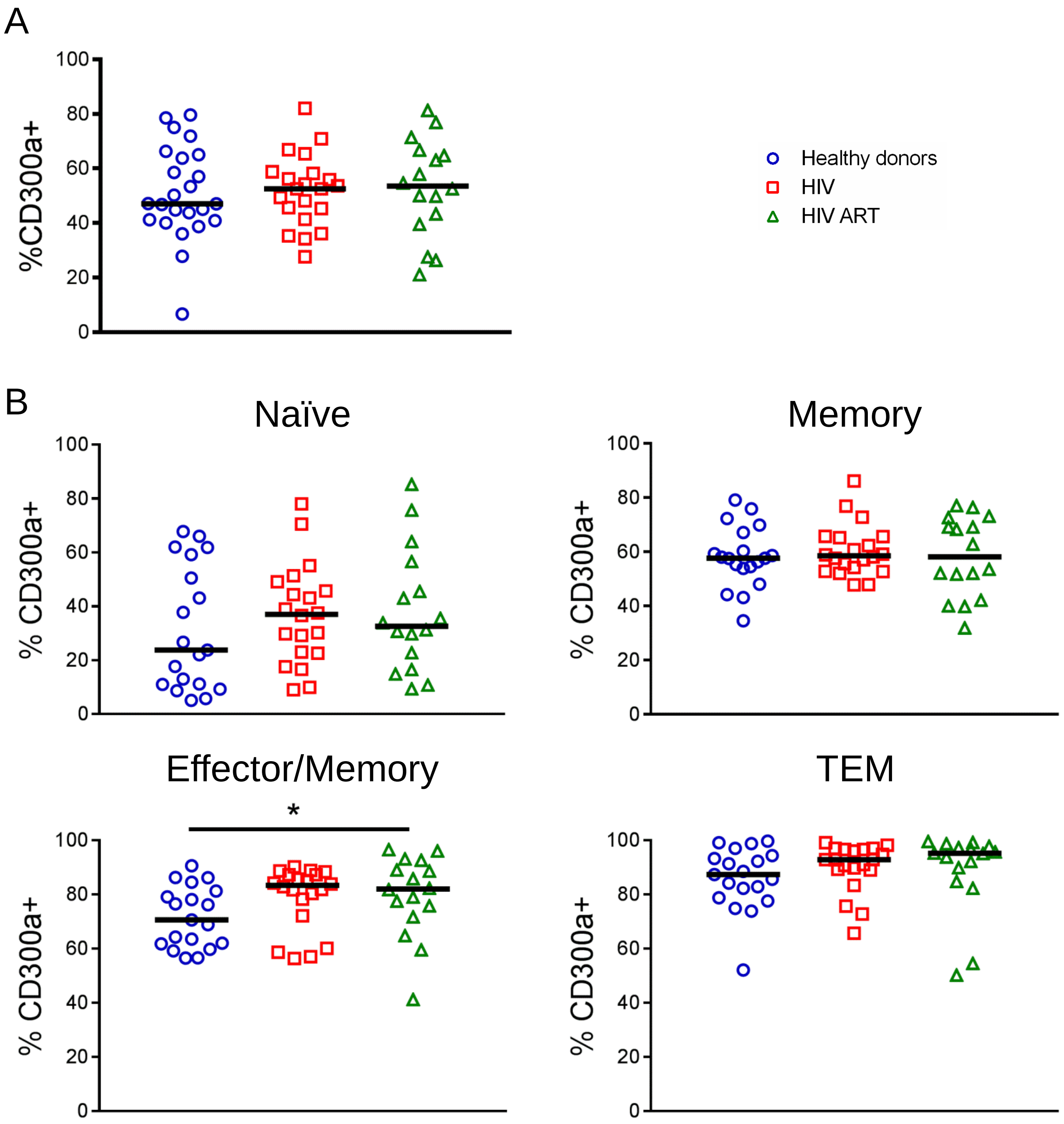

Supplementary Figure 2. Percentage of CD300a+ cells on CD4+ T cells from healthy donors and HIV-1 infected patients. Dot plots showing (A) the percentage of CD300a+ cells in total CD4+ T cells and (B) within CD4+ T cell subsets from healthy donors, naïve for cART (HIV) and patients on cART (HIV ART). Each dot represents a subject and the median is shown. *p<0.05.

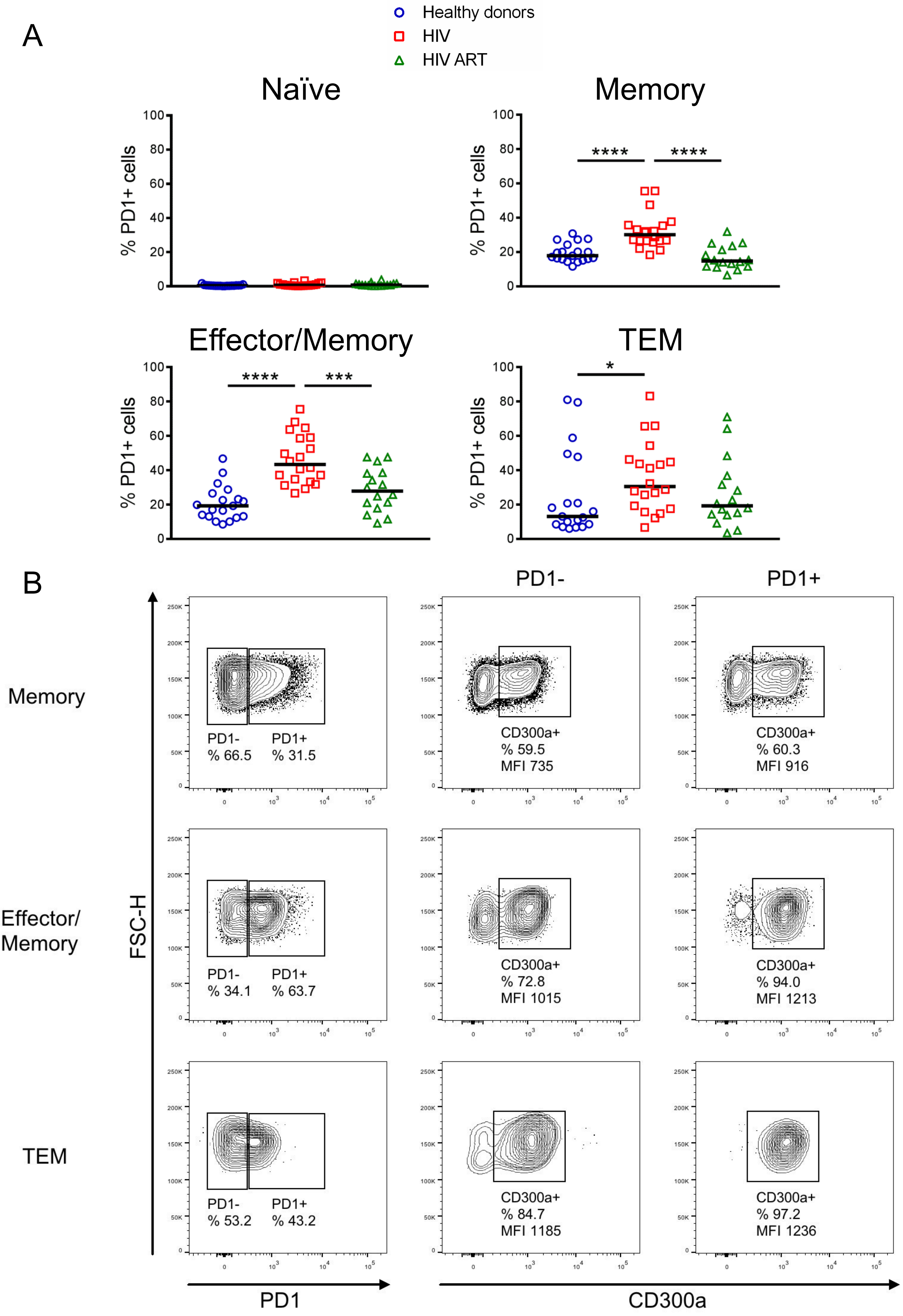
A
Healthy donors
HIV
HIV ART
Naïve
Memory
Effector/Memory
TEM
% PD1+ cells
100
80
60
40
20
0
****
****
***
*
B
PD1-
PD1+
Memory
Effector/
Memory
TEM
FSC-H
PD1
CD300a
PD1-
% 66.5
PD1+
% 31.5
CD300a+
% 59.5
MFI 735
CD300a+
% 60.3
MFI 916
PD1-
% 34.1
PD1+
% 63.7
CD300a+
% 72.8
MFI 1015
CD300a+
% 94.0
MFI 1213
PD1-
% 53.2
PD1+
% 43.2
CD300a+
% 84.7
MFI 1185
CD300a+
% 97.2
MFI 1236

Supplementary Figure 3. Percentage of PD1+ and PD1- cells within CD4+ T lymphocytes. (A) Dot plots representing the percentage of PD1+ cells within CD4+ T cells from healthy donors, cART naïve (HIV) and patients on cART (HIV ART). Each dot represents a subject and the median is shown. (B) Contour plots showing the percentage of PD1+ and PD1- cells, and the percentage of CD300a+ cells within PD1+ and PD1- cells on CD4+ T lymphocytes. Data from a representative untreated HIV-1 infected patient is shown. *$p<0.05$, *** $p<0.001$, **** $p<0.0001$.

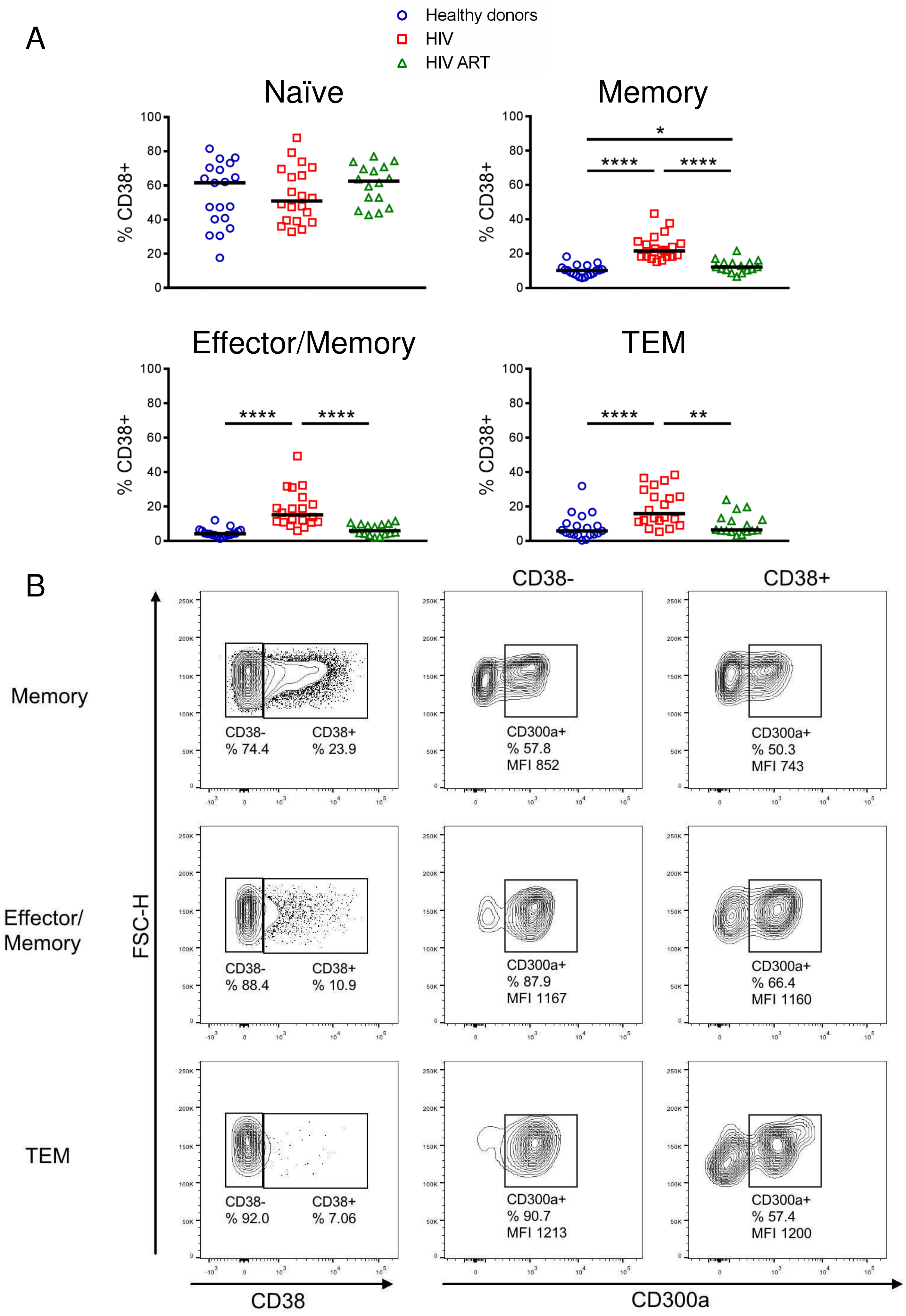
A
Healthy donors
HIV
HIV ART
Naïve
Memory
Effector/Memory
TEM
% CD38+
*
****
**
B
CD38-
CD38+
Memory
Effector/
Memory
TEM
FSC-H
CD38-
% 74.4
CD38+
% 23.9
CD300a+
% 57.8
MFI 852
CD300a+
% 50.3
MFI 743
CD38-
% 88.4
CD38+
% 10.9
CD300a+
% 87.9
MFI 1167
CD300a+
% 66.4
MFI 1160
CD38-
% 92.0
CD38+
% 7.06
CD300a+
% 90.7
MFI 1213
CD300a+
% 57.4
MFI 1200
CD38
CD300a

Supplementary Figure 4. Percentage of CD38+ and CD38- cells within CD4+ T lymphocytes. (A) Dot plots representing the percentage of CD38+ cells within CD4+ T cells from healthy donors, cART naïve (HIV) and patients on cART (HIV ART). Each dot represents a subject and the median is shown. (B) Contour plots showing the percentage of CD38+ and CD38- cells, and the percentage of CD300a+ cells within CD38+ and CD38- cells on CD4+ T lymphocytes. Data from a representative untreated HIV-1 infected patient is shown. *p<0.05, ** p<0.01, **** p<0.0001.

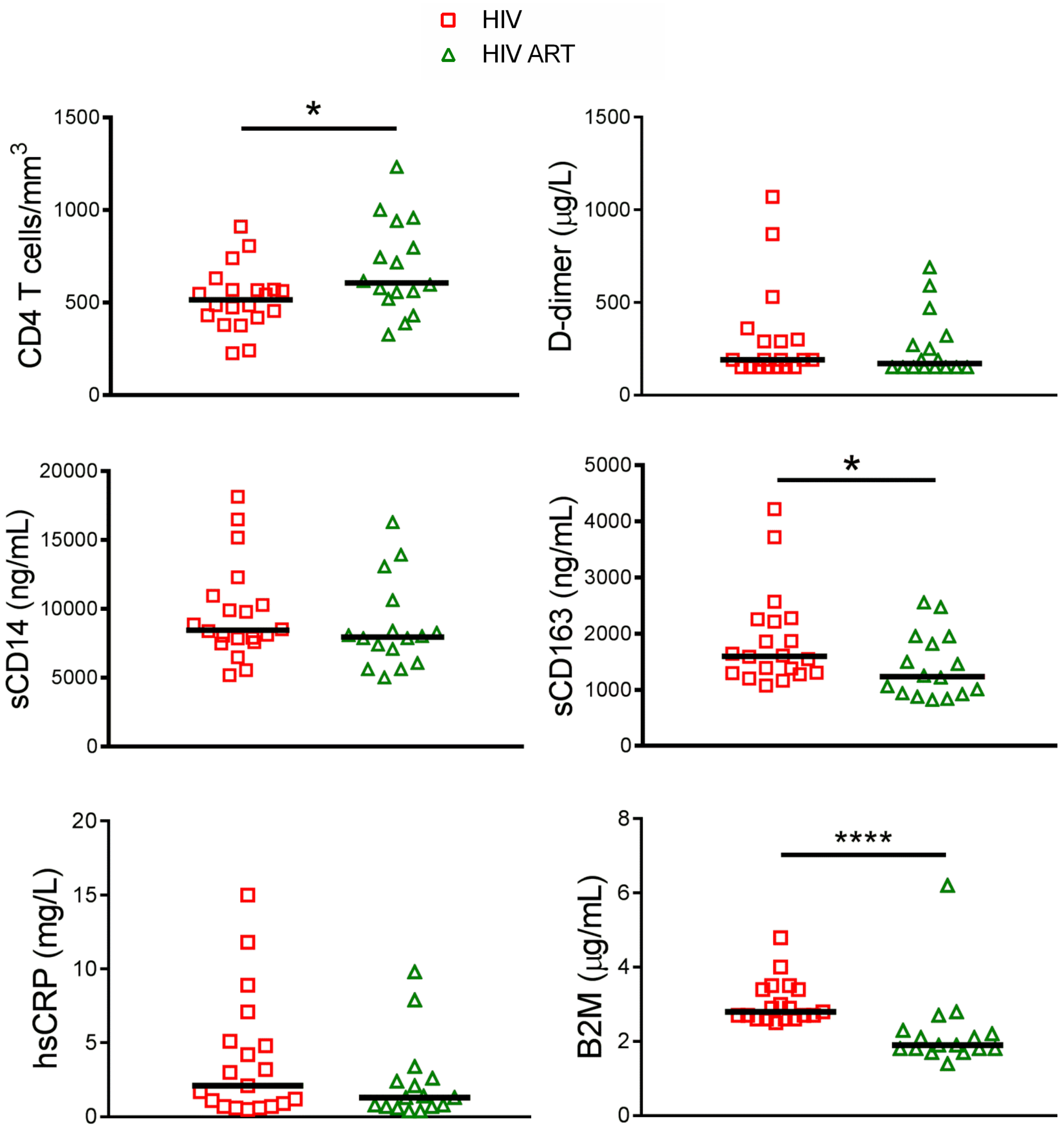

Supplementary Figure 5. Markers of HIV-1 disease progression in untreated and patients on cART (HIV ART). Dot plots representing CD4+ T cell count and plasma levels of D-dimer, soluble CD14 (sCD14), soluble CD163 (sCD163), high sensitive C-reactive protein (hsCRP) and beta-2-microglobulin (B2M) from cART naïve and patients on cART (HIV ART). Each dot represents a subject and the median is shown. *p<0.05, **** p<0.0001.

S1 Table. Clinical data of cART naïve HIV-1-infected patients.

**PATIENTS**	**SEX**	**AGE**	**CD4**	**VL**
28898	MAN	28	910	199000
29589	MAN	36	569	120000
25998	MAN	33	562	12900
29208	MAN	49	418	51900
27315	MAN	46	454	233000
28285	MAN	28	376	65400
28178	MAN	23	241	268000
26183	MAN	24	568	20600
27213	MAN	30	547	22800
28109	MAN	22	806	61400
28547	MAN	25	486	11400
29384	MAN	29	484	72600
30385	MAN	47	226	47500
27090	MAN	20	630	97600
29452	MAN	35	543	43400
30995	MAN	32	431	75700
27594	MAN	30	378	93200
26558	MAN	27	739	9350
25966	WOMAN	26	473	32400
25909	MAN	20	567	21700

S2 Table. Clinical data of cART treated HIV-1-infected patients

**PATIENTS**	**SEX**	**AGE**	**cART years**	**CD4 T count**	**VL**
30318	MAN	31	3	519	<20
30135	MAN	45	9	596	<20
29987	MAN	69	15	387	<20
29984	MAN	45	7	958	<20
29971	WOMAN	28	2	796	<20
29970	MAN	68	16	560	<20
29969	WOMAN	53	12	744	<20
29965	WOMAN	35	2	1001	<20
29935	MAN	62	7	555	<20
29925	MAN	39	12	577	<20
29919	MAN	60	24	325	<20
29909	MAN	52	3	616	<20
29895	MAN	41	5	941	<20
29879	MAN	58	12	1233	<20
29862	MAN	32	3	429	<20
29854	MAN	28	2	716	<20
